# Dedifferentiation of *Arabidopsis thaliana* cells is accompanied by a strong decrease in RNA polymerase II transcription activity and poly(A+) RNA and 25S rRNA eradication from the cytoplasm

**DOI:** 10.1007/s00709-014-0700-6

**Published:** 2014-09-24

**Authors:** Konrad Dełeńko, Janusz Niedojadło, Agata Łabędzka, Ewa Wiśniewska, Elżbieta Bednarska-Kozakiewicz

**Affiliations:** 1 Department of Cell Biology, Faculty of Biology and Environment Protection, Nicolaus Copernicus University, Gagarina 9, 87-100 Toruń, Poland; 2 Department of Clinical Pathomorphology, Ludwik Rydygier Collegium Medium Bydgoszcz, Nicolaus Copernicus University in Toruń, Skłodowskiej-Curie 9, 85-094 Bydgoszcz, Poland

**Keywords:** Dedifferentiation, RNA polymerase II transcription, Poly(A+) RNA, Protoplasts, *Arabidopsis thaliana*

## Abstract

The mechanisms of plant cell dedifferentiation and the acquisition of totipotency are poorly understood. One of the methods to induce the dedifferentiation process in plant cells is simple and requires the removal of the cell wall. After cell wall removal in protoplasts, large-scale chromatin decondensation is observed (Tessadori et al. in J Cell Sci 120:1200–1208, 2007). Here, we show that in *Arabidopsis thaliana* protoplasts, despite chromatin decondensation, RNA polymerase II transcriptional activity is reduced. The subsequent investigated stages displayed a clear decrease in the quantity of 25S ribosomal RNA (rRNA) first and then poly(A+) RNA, particularly in the cytoplasm. Therefore, the reduced transcription activity and the removal of these RNA transcripts from the cytoplasm is a crucial process in obtaining totipotency in plant cells. After the cytoplasm cleaning of transcripts derived from mesophyll cells, we observed the resynthesis of these RNAs. An increase in the amount of examined molecules to a level similar to that in differentiated mesophyll cells precedes the divisions of already undifferentiated cells. In this work, we show changes in RNA polymerase II transcription dynamics and the quantity of poly(A+) RNA and 25S rRNA during dedifferentiation and re-entry into the cell cycle.

## Introduction

Dedifferentiation is a remarkable process in which a specialised cell becomes a simpler, unspecialised cell. In plant cells, dedifferentiation can occur after enzymatic degradation of the cell wall, which leads to the formation of protoplasts. The most common sources of protoplasts are leaf mesophyll cells (Jiang et al. 2013). After the degradation of their cell wall, these specialised mesophyll cells become unspecialised, totipotent protoplasts, which can regenerate the entire plant with an intermediate microcalli stage in the appropriate conditions (Takebe et al. 1971; Guri et al. 1987; Kim and Lee 1996; Chupeau et al. 2013). The transition from leaf mesophyll to protoplast requires many changes in cellular metabolism as a result of adaptation to the new stressful environmental conditions. One of the first changes is an alteration in cell nucleus architecture. The *Arabidopsis thaliana* interphase cell nucleus has heterochromatin organised in so-called chromocenters, which contain heavily methylated, mostly repetitive DNA sequences (Fransz et al. 2002). Freshly isolated protoplasts from *A. thaliana* have a decrease in the number and size of chromocenters as a consequence of chromatin decondensation. However, despite the chromatin decondensation, epigenetic markers of heterochromatin (histone H3K9 dimethylation and 5-methylcytosine level) remain unchanged (Tessadori et al. 2007). An analysis of *Nicotiana tabacum* protoplasts and cultured cells (derived from protoplasts) showed changes in cell nucleus architecture similar to *A. thaliana*, but in this system, two distinct phases of chromatin decondensation were observed. The first phase occurs in freshly isolated protoplasts, and the second one occurs when cultured cells re-enter the cell cycle and progress into the S-phase (Zhao et al. 2001). However, whether such a sudden change affects the global transcription level and further steps of gene expression is unclear.

Notably, many features of protoplasts have been confirmed in animal stem cells due to the similarity in their features (Grafi et al. 2011). The decondensation state of chromatin is well known in undifferentiated animal embryonic stem cells (ESCs), and a less condensed chromatin is considered a feature of pluripotent cells, which makes them different from somatic and differentiated cells. A study on mouse ESCs showed that the genome of these cells is transcriptionally globally hyperactive, which indicates that a large part of the genome is expressed at a low level. In ESCs, normally silent regions (such as major and minor satellite repeats, LINEs, SINEs and retrotransposons) are expressed, and tissue-specific gene transcripts are present sporadically at low levels (Efroni et al. 2008). In human ESCs, most promoters of protein-coding genes (approximately 75 %) are occupied by the initiation form of RNA polymerase II (RNA POL II), but only half of them are expressed at a detectable level (Guenther et al. 2007). RNA POL II could be arrested on the promoter, in the initiation or early elongation step, due to transcriptional pausing or abortive initiation (Dvir 2002; Nechaev and Adelman 2011; Adelman and Lis 2012). Therefore, changes in chromatin structure and RNA POL II activity are a characteristic of totipotent animal cells.

RNA POL II transcription is regulated i.a. by *cis*-acting regulatory elements, transcription factors (TFs) and Mediator complex (Kadonaga 2004; Yamaguchi-Shinozaki and Shinozaki 2005; Poss et al. 2013). During exposure to various stress conditions and in response to hormone, changes occur in transcript abundance of several genes encoding Mediator subunits (Pasrija and Thakur 2012). Changes in Mediator subunit composition have an effect on the gene expression profile of these cells. *A. thaliana* recruitment of RNA POL II to some of cold-regulated CBF-responsive genes and their expression induced by low temperature depends on three Mediator complex subunits (MED16, MED2 and MED14) (Hemsley et al. 2014). After the degradation of the cell wall, many TFs and Mediator subunit transcripts are also deregulated in protoplasts due to stress experienced by these cells (Chupeau et al. 2013). However, nothing is known about how these changes affect RNA POL II transcription in these cells.

The steps of gene transcription (initiation, elongation and termination) are strictly associated with the phosphorylation pattern of the RNA POL II C-terminal domain (CTD) of its largest subunit Rpb1 (Hsin and Manley 2012). The CTD domain of RNA POL II consists of 26 (*Saccharomyces cerevisiae*) to 52 (*Homo sapiens*) tandem repeats of the consensus sequence Y^1^S^2^P^3^T^4^S^5^P^6^S^7^ (Hsin and Manley 2012; Zhang et al. 2012). RNA POL II binds to the promoter with an unphosphorylated CTD domain; next, at transcription initiation, Ser5 and Ser7 are phosphorylated. During elongation, the phosphate groups from Ser5 and Ser7 are gradually removed by phosphatases, while Ser2 is phosphorylated. Near termination, the dephosphorylation of Ser2 occurs, and unphosphorylated CTD facilitates the release of RNA POL II from the DNA, allowing RNA POL II to be recycled and begin a new round of transcription (Buratowski 2009; Hsin and Manley 2012; Zhang et al. 2012). These features of the CTD could be used to determine the state of RNA POL II in the cell nucleus and to study transcriptional activity changes in time.

In this study, we analysed the global transcription level of RNA polymerases I and II through localisation and quantitative measurement of the elongation form of RNA POL II (RNA POL II EF) and poly(A+) RNA and 25S ribosomal RNA (rRNA) transcripts in *A. thaliana* cells undergoing dedifferentiation.

## Materials and methods

### In vitro culture, protoplast isolation and culturing

*A. thaliana* Col-0 seeds were washed in 70 % ethanol for 2 min, sterilised in 6 % calcium hypochlorite solution for 13 min and washed 10 times for 3 min in sterile water. Then, the seeds were sown in 75 % Murashige and Skoog medium supplemented with 0.7 % (*w*/*v*) agar. The growth conditions were continuous light with 45 μmol m^−2^ s^−1^ irradiance and an ambient temperature of 24 °C. Protoplasts were isolated from 14-day-old plantlets. The isolation and culturing of protoplasts and all necessary solutions were prepared according to the protocol of Chupeau et al. (2013). For every experiment conducted in this research, we used leaf mesophyll cells, freshly isolated protoplasts and cells derived from protoplasts (CDP) cultured up to 120 h.

### Fixation and cell membrane permeabilisation

All plant material used for fluorescence in situ hybridisation (FISH) and immunofluorescence analysis was fixed in 4 % formaldehyde (Polysciences, Inc, Warrington, PA, USA) diluted in PIPES (Sigma-Aldrich, St. Louis, MO, USA) for 1 h (leaves were placed in a vacuum for 1 h to remove air from the intercellular spaces). After fixation, the material was washed five times in PBS for 3 min. In the case of leaf mesophyll cells, we modified the protocol of nuclei isolation (Tirichine et al. 2009) to obtain single cells besides isolated nuclei. For this purpose, we gently homogenised plantlets in Potter homogeniser. For permeabilisation of the cell membrane, we used increasing dilutions of Triton X-100 in phosphate-buffered saline (PBS) from 1:5000 for protoplasts to 1:2000 for CDP cultured for 5 days (120 h). After treatment with Triton X-100, the examined material was washed three times for 5 min in PBS and used for FISH and immunofluorescence analysis. Next, all procedures were conducted in 1.5-ml Eppendorf tubes, and when solutions were changed, the samples were centrifuged for 3.5 min (0.1 × *g* for protoplasts and CDP, 0.3 × *g* for isolated nuclei).

### Fluorescence in situ hybridisation (FISH)

FISH was conducted for a minimum of 16 h (with a 1-h pre-hybridisation step in the same buffer) using hybridisation buffer with the following composition: 50 % (*v*/*v*) hybridisation buffer (Sigma-Aldrich, St. Louis, MO, USA), 30 % (*v*/*v*) formamide (Sigma-Aldrich, St. Louis, MO, USA) and 20 % (*v*/*v*) H_2_O. For the analysis of 25S rRNA localisation and amount, we used the oligo probe 5′Cy3-AGCTACTAGATGGTTCGATTAGTCTTTC3′; the hybridisation step was performed at 37 °C. Transcripts with a poly(A+) tail were visualised using a 30-nt Cy3-oligothymidyne probe, and FISH was performed at 26 °C. The post-hybridisation steps in the case of 25S rRNA were washes in 4×, 2× and 1× the concentrations of SSC for 5 min each; for poly(A+) RNA, the FISH materials were washed three times in 4× SSC for 5 min and once in 2× SSC. For FISH double-labelling (U2 small nuclear RNA (snRNA) and poly(A+) RNA), both probes were applied simultaneously in the hybridisation medium, and hybridisation was conducted at 30 °C using antisense U2 snRNA 5′ rhodamine green-ATATTAAACTGATAAGAACAGATACTACACTTG. Control reactions were conducted without oligo probes. DNA was visualised by 4,6-diamidino-2-phenylindole (DAPI; Sigma-Aldrich, St. Louis, MO, USA) staining in all reactions.

### Immunolocalisation of phosphorylated serine-2 in the CTD domain of RNA POL II

After permeabilisation with Triton X-100, isolated nuclei, protoplast suspensions and CDP were incubated with a rat anti-phosphoserine-2 primary antibody IgG against the CTD heptapeptide repeat (ChromoTek, Martinsried, Germany) diluted 1:300 in 1 % BSA in PBS for at least 16 h at 4 °C. Next, the examined materials were washed three times in PBS and incubated with a goat anti-rat secondary antibody IgG labelled with Alexa Fluor 488 (Molecular Probes, Inc., Eugene, OR, USA) and diluted 1:500 for 1 h at 35 °C. The secondary antibody was washed three times for 5 min in PBS, and the samples were stained with DAPI (Sigma-Aldrich, St. Louis, MO, USA). The control reactions were performed without primary antibody.

### Microscopy analysis, quantitative measurement of fluorescence and statistical analysis

Protoplasts and cultured cells were observed using a Nikon TS-100 light-inverted microscope equipped with a PRIOR Lumen 200 fluorescent lamp and a Nikon DS-U3 digital camera. The results of FISH and immunolocalisation were analysed with a Nikon PCM-2000 confocal microscope and fluorescence-inverted Nikon Eclipse TE 2000-E microscope. A 100× (numerical aperture, 1.3) Plan Fluor DIC H/N2 oil immersion lens was used. For each material (mesophyll-isolated nuclei and cells, protoplasts and CDP) and each reaction, we obtained three-dimensional optical sections with a 0.5-μm step interval from 22 to 46 cells, depending on the stage. The obtained data were corrected for background autofluorescence determined based on negative control signal intensities. All measurements were conducted at the same magnification, area and time of laser scanning. For the image processing and analysis, NIS-Elements F 3.2 (Laboratory Imaging, Praha, Czech Republic), EZ Viewer software package (Nikon Europe BV, Badhoevedorp, Netherlands) and ImageJ (Schneider et al. 2012) software were used. For signal evaluation, Cell Statistical Analyser 1.0.1 (Department of Cell Biology, Nicolaus Copernicus University, Toruń, Poland) software was used. The signal intensity per cubic micrometre is expressed in arbitrary unit of fluorescence intensity (a.u.). Statistical analysis was performed using PAST (Hammer et al. 2001) and Microsoft Excel (Microsoft, Redmond, WA, USA). To compare all groups and to determine if there were any significant differences between them, a non-parametric Kruskal-Wallis test was used. To test between which group differences exist, a Mann-Whitney *U* test with Bonferroni correction was used.

## Results

### Protoplasts and CDP culture

**Fig. 1:**
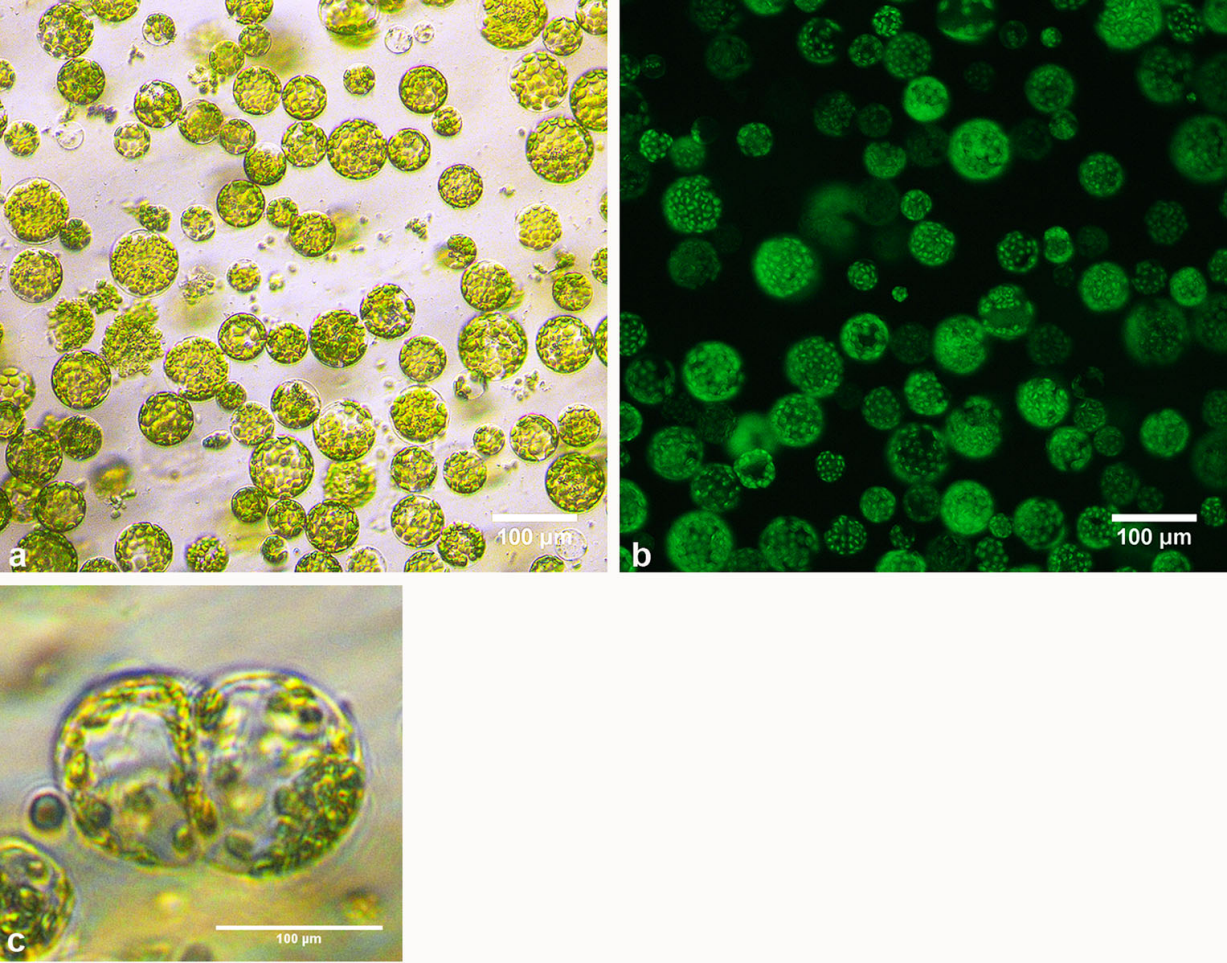
Micrographs of protoplast and dividing cells in culture. **a** Freshly isolated protoplasts, **b** the same cells stained with fluorescein diacetate (FDA) under blue light. **c** Divided CDP after 120 h of culture

Protoplasts are a very convenient and reproducible model to study the dedifferentiation process. From each mesophyll protoplast isolation (Fig. 1a), we obtained approximately 75–80 % viable cells (Fig. 1b). Because protoplasts very soon regenerate their cellulose cell wall, cells cultured from 24 to 120 h were called cells derived from protoplasts (CDP). We observed the first cell divisions between 72 and 96 h; however, we conducted our analysis on CDP cultured for 120 h because more divided cells were evident at this stage (Fig. 1c). After 120 h of culture, approximately 40 % of cells in the CDP population were dead, 45–50 % had not divided but were viable and 10–15 % had divided, depending on the isolation. During culture, we observed the gradual disappearance of chlorophyll, so structures similar to chloroplasts in later stages were called plastids (Fig. 1c). Using this well-established cell culture method, we performed an analysis of the amount and distribution of RNA POL II, poly(A+) RNA and 25S rRNA in protoplasts and cells cultured for 24, 72 and 120 h.

### Distribution and level changes of RNA POL II during dedifferentiation

**Fig. 2:**
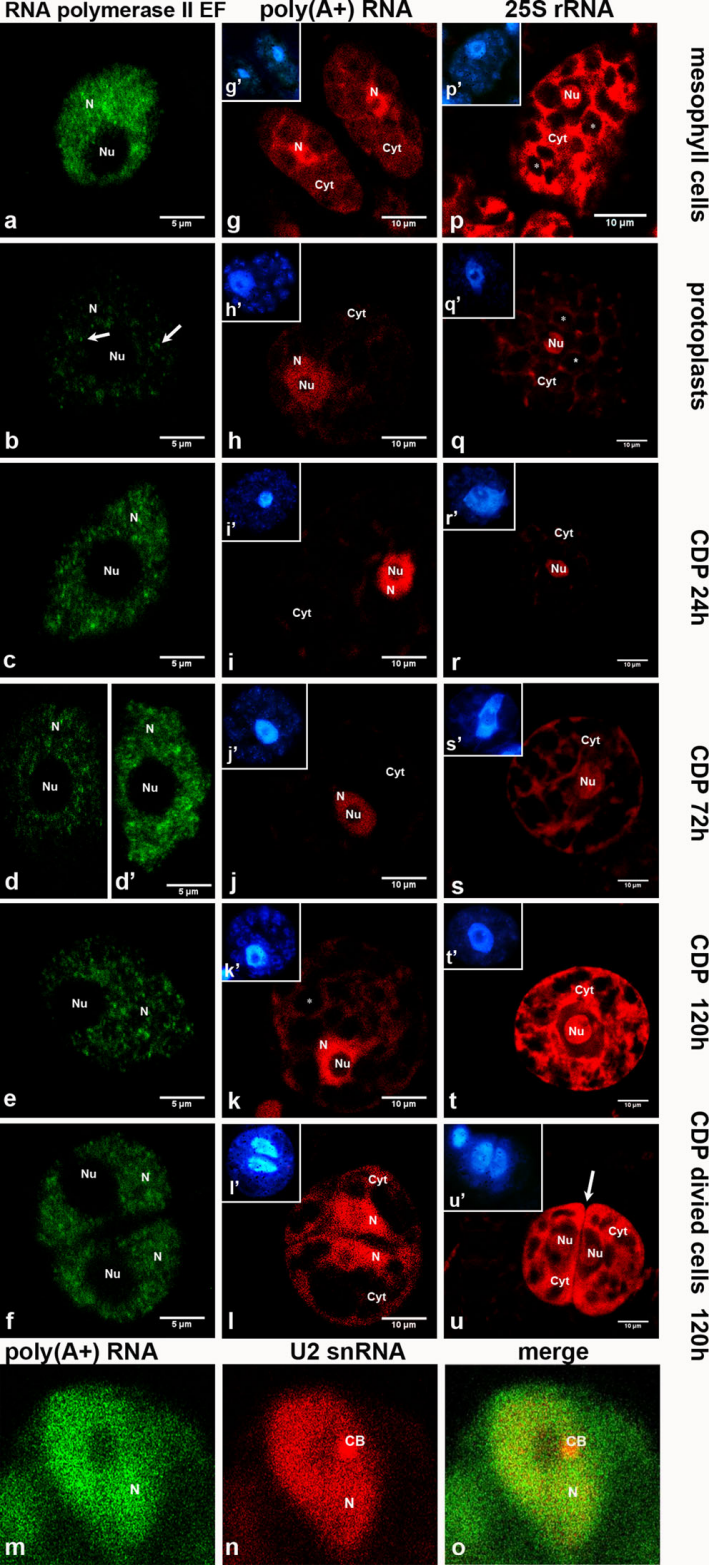
**a**–**f** Localisation of RNA polymerase II elongation form, **g**–**l** poly(A+) RNA, **p**–**u** 25S rRNA transcripts and **m**–**o** co-localisation of poly(A+) RNA with U2 snRNA in leaf mesophyll cells, protoplasts and CDP of *Arabidopsis thaliana*. **a** Nucleus isolated from leaf mesophyll cells. A homogenous fluorescence signal occurs in the entire nucleoplasm, and the nucleolus is devoid of signal. **b** Nucleus from a protoplast immediately after isolation (0 h). A weak fluorescence signal is visible in the form of different sizes of foci (indicated by an *arrow*). **c** Cell nucleus at 24 h after isolation. RNA POL II EF foci occur in the entire nucleoplasm. **d** CDP after 72 h showing a cell with weak staining similar to protoplasts. **d**′ Cell nucleus 72 h after isolation and right before cell division. The distribution of RNA POL II EF is comparable with that observed in nuclei isolated from leaf mesophyll cells. **e** Nucleus from a non-divided cell at 120 h after isolation. Easily distinguishable RNA POL II EF foci can be observed. **f** Two daughter nuclei after cell division show RNA POL II EF distributed homogenously. The fluorescence is weaker than in the 72-h CDP. **g**–**l** Localisation of poly(A+) RNA transcripts by FISH. **g** Leaf mesophyll cells show strong, homogenous labelling of the nucleoplasm; a weaker signal is observed between chloroplasts in the cytoplasm. **h** Protoplast with poly(A+) RNA distribution similar to mesophyll cells. **i** CDP cultured for 24 h. Staining is increased in the nucleus and decreased in the cytoplasm compared to a protoplast. A few aggregates are visible in the cytoplasm, while most of the signal is concentrated in the nucleus. **j** CDP after 72 h have a homogenous signal that is weaker than in the 24-h cultured CDP and is observed mainly in the cell nucleus. **k** A CDP cultured for 120 h is not yet divided and shows a strong homogenous signal in the nucleus; in the cytoplasm, the signal is diffused between cell compartments. **l** Divided cells after 120 h of culture show strong labelling in the nucleus. A newly synthesised cell wall is visible between cells. **g**′–**l**′ DAPI staining (*blue*) of cells in g–l. **m**–**o** Co-localisation of poly(A+) RNA and U2 snRNA (*red*) in nuclei of CDP cultured for 120 h. **m** Poly(A+) RNA signal occurs both in the nucleoplasm and cytoplasm. **n** The U2 snRNA signal is present mainly in the nucleus. In the nucleus, U2 snRNA is distributed homogenously in the nucleoplasm with signal aggregation representing a Cajal body (CB). **o** A merge shows no co-localisation of poly(A+) RNA and U2 snRNA in CBs. **p**–**u** Localisation of 25S rRNA transcripts by FISH. **p** Cell from leaf mesophyll tissue with fragments of adjacent cells. A strong, homogenous signal is observed in the nucleolus and in the spaces between chloroplasts. **q** Protoplast with decreased fluorescence intensity in the entire cell compared to a mesophyll cell with no changes in the distribution pattern. **r** A very weak fluorescence is observed in the cytoplasm in CDP after 24 h of cell wall removal. **s** CDP cultured for 72 h shows visible large nucleolus in the centre of the cell. The signal re-appears in the cytoplasm and occurs in spaces between plastids and under the cell membrane. **t** Non-divided CDP after 120 h of culture. A large, strongly labelled nucleolus is visible in the centre of the cell. A very intense and homogenous signal is evident in the cytoplasm. **u** Two divided cells after 120 h of culture. A visible, strong, homogenous signal in the cytoplasm is especially concentrated near the new cell wall (indicated by an *arrow*), and the signal is diffused in nucleoli and dispersed in the nucleoplasm. **p**′–**u**′ DAPI staining (*blue*) of cells in p–u. *N* nucleoplasm, *Nu* nucleolus, *Cyt* cytoplasm, * chloroplasts/plastids

**Fig. 3:**
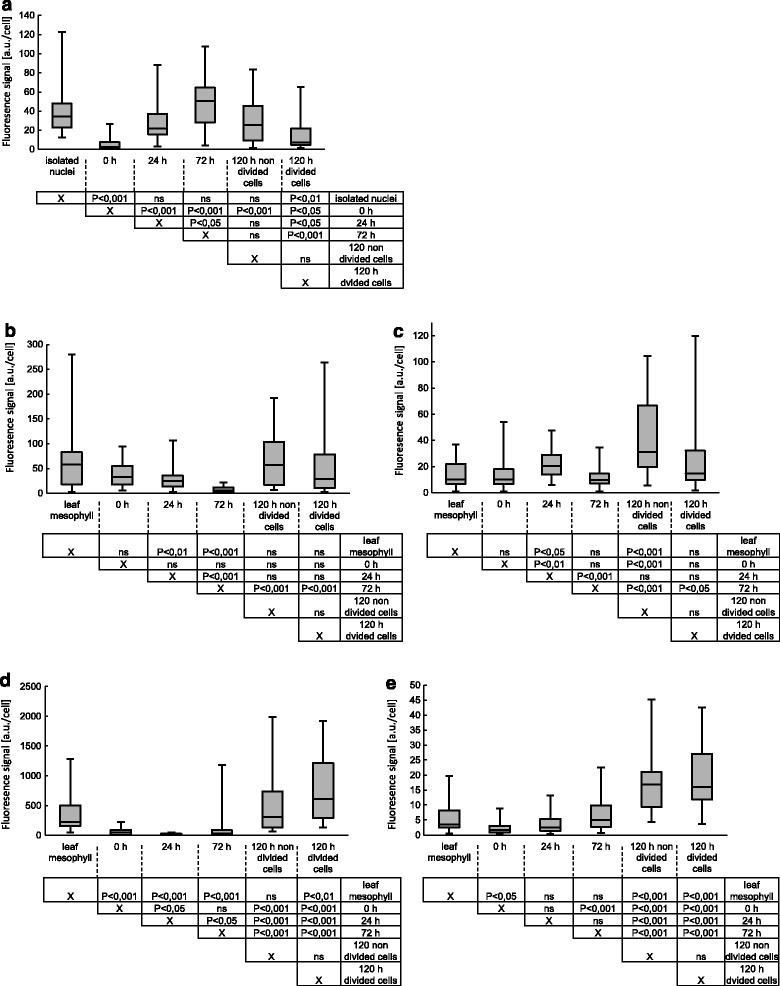
Box plots showing quantitative analysis of examined molecules at each stage. **a** Amount of RNA POL II EF. **b** Cytoplasmic amount of poly(A+) RNA. **c** Poly(A+) RNA in nuclei. **d** Cytoplasmic amount of 25S rRNA transcripts. **e** 25S rRNA transcripts in nucleoli. In the box plot, *black lines* and *boxes* represent the median and first and third quartiles of the values, respectively; *whiskers* extend to the minimum and maximum values. Significant differences between groups, with *P* value (starting at *P* < 0,05), are shown in the diagrams under the plots. *ns* indicates non-significant differences between the groups

In all tested cells among every stage, fluorescence indicating the presence of RNA POL II EF was observed only in the nucleoplasm and not the nucleolus in the cell nucleus; the signal was undetectable in the cytoplasm (Fig. 2a–f). In nuclei isolated from a leaf mesophyll tissue, RNA POL II EF was present homogeneously and in a high amount in the entire nucleoplasm (Fig. 2a). Compared to nuclei isolated from a leaf mesophyll tissue, in the protoplast nucleoplasm, the signal was low and was distributed in a punctate pattern represented by foci of different sizes (Fig. 2b). Our quantitative analysis demonstrated that after protoplasting, the amount of RNA POL II EF strongly decreased by approximately 8.5-fold compared to isolated nuclei from mesophyll cells (Fig. 3a). After 24 h of culture, the fluorescence in CDP nuclei increased compared to protoplasts and occurred in a form of foci of higher intensity (Fig. 2c). A quantitative fluorescence signal evaluation shows that after 24 h of culture, the amount of RNA POL II EF increased and reached a level observed in nuclei isolated from mesophyll cells (Fig. 3a). In CDP cultured for 72 h, we observed two types of cell populations that had different signal distribution patterns (Fig. 2d–d′). One population had a signal distribution similar to the 24-h CDP (Fig. 2d), and the second one had fluorescence more similar to that observed in nuclei isolated from mesophyll cells with visible larger aggregates (Fig. 2d′). However, we observed more cells from the second population where fluorescence was more intense. Hence, the amount of RNA POL II EF increased compared to the 24-h CDP and was similar to mesophyll cells (Fig. 3a). In non-divided cells (Fig. 1e) after 120 h of culture, the fluorescence was similar to the 24-h CDP, and easily distinguishable foci were observed in the nucleus. Non-divided CDP after 120 h had a level of RNA POL II EF similar to the 72-h CDP (Fig. 3a). Among the divided cells (Fig. 2f), the signal decreased significantly compared to CDP cultured for 72 h, and a homogeneous signal predominated in the nuclei. The quantification of fluorescence per nucleus indicated that RNA POL II EF level decreased significantly in divided cells compared to the 72-h CDP. Between the divided and non-divided cells, the RNA POL II EF level was comparable, and no statistically significant differences were found between these two groups.

### Poly(A+) RNA localisation and level changes during dedifferentiation

After poly(A+) RNA detection in leaf mesophyll cells, a signal was visible in the nucleus and cytoplasm (Fig. 2g). In cells of every analysed stage, the signal was not present in nucleoli. In the nucleus, the signal was strong and homogenous. In the cytoplasm, the signal was weaker than in the nucleus and filled the spaces between chloroplasts.

In protoplasts, the signal pattern was very similar to mesophyll cells (Fig. 2h). Despite a large decrease in RNA POL II quantity over this stage, cytoplasmic (Fig. 3b) and nuclear (Fig. 3c) levels of poly(A+) RNA did not decrease significantly. However, a lower concentration of poly(A+) RNA can be observed in both these compartments in the micrographs, and this result may be related to a noticeable increase in nuclear/whole cell volume after protoplasting.

After 24 h of CDP culture, a strong homogenous signal was observed in cell nuclei, and the fluorescence decreased in the cytoplasm (Fig. 2i). The cytoplasmic amount of poly(A+) RNA transcripts did not change compared to protoplasts but was lower than in mesophyll cells (Fig. 3b), and the quantity of these transcripts increased in nuclei compared to protoplasts (Fig. 3c).

After 72 h in CDP, the fluorescence signal decreased in the entire cell and occurred mainly in the cell nucleus; the cytoplasmic signal was almost not visible (Fig. 2j). The cytoplasmic (Fig. 3b) and nuclear (Fig. 3c) amount of poly(A+) RNA decreased significantly and was the lowest among all tested stages.

After 120 h of culture, in non-divided CDP (Fig. 2k) and divided CDP (Fig. 2l), poly(A+) RNA transcripts were observed again in the cytoplasm with a distribution pattern similar to leaf mesophyll cells or protoplasts; in both types of CDPs, the nuclei were the most strongly labelled compartment.

In both non-divided and divided CDPs, the poly(A+) RNA level in the cytoplasm increased compared to the 72-h CDP and recovered to an amount similar to mesophyll cells (Fig. 3b). However, the non-divided cells had significantly more poly(A+) in the cell nuclei compared to leaf mesophyll cells (Fig. 3c).

Recently, Kołowerzo et al. (2009) showed that poly(A+) RNA transcripts accumulate in Cajal bodies (CBs) of diplotene larch microsporocytes after stages of increased transcription. Therefore, we determined whether poly(A+) RNA accumulation in CBs also occurs in the stages we studied. We examined the co-localisation of poly(A+) RNA (Fig. 2m) and U2 snRNA (Fig. 2n) (known as a marker of CBs in plant cells) in nuclei isolated from mesophyll cells, protoplasts and CDP. In any of the tested stages, we did not observe the accumulation of poly(A+) RNA in CBs. However, a homogenous fluorescence signal of poly(A+) RNA usually overlapped with U2 snRNA-staining bodies (Fig. 2o).

### 25S rRNA localisation and level fluctuation during dedifferentiation

After FISH treatment for 25S rRNA transcripts, in all tested cells among every stage, the fluorescence was present in nucleoli and at a lower level in the nucleoplasm and cytoplasm (Fig. 2p–u). The nucleoplasm in every stage was the weakest stained cell compartment. Between stages, the most variable signal distribution pattern occurred in the cytoplasm.

In leaf mesophyll cells, fluorescence was present in nucleoli and the cytoplasm (Fig. 2p). The fluorescence in nucleoli was intense and homogenous with a few small areas that showed weaker labelling. In the cytoplasm, the signal occurred in the spaces between chloroplasts.

After removing the cell wall, we observed a significant decrease in fluorescence intensity in whole cells, nucleoli and the cytoplasm in protoplasts (Fig. 2q). A quantitative analysis showed that the level of 25S rRNA transcripts was approximately sixfold lower in the cytoplasm of protoplasts compared to mesophyll cells (Fig. 3d), and the 25S rRNA level was twofold lower in nucleoli (Fig. 3e).

At 24 h after cell wall removal, 25S rRNA transcripts were observed in a very small area of the CDP cytoplasm in the form of single aggregates (Fig. 2r). The cytoplasmic amount of 25S rRNA after 24 h of culture was the lowest among all tested stages (Fig. 3d). Although the cytoplasmic rRNA level was the lowest, the amount of rRNA in nucleoli increased 1.4-fold compared to protoplasts (Fig. 3e).

After 72 h of culture, fluorescence was observed in CDP nucleoli, and in the cytoplasm, 25S rRNA transcripts began to be visible again at a high level (Fig. 2s). The CDP at this stage had a significantly higher (approximately 6.5-fold higher) level of cytoplasmic 25S rRNA than in the 24-h CDP (Fig. 3d). The amount of 25S rRNA in nucleoli was similar to CDP after 24 h of culture (Fig. 3e).

After 120 h of culture, the fluorescence signal was very strong in nucleoli of non-divided CDP and was present in a pattern similar to that observed in mesophyll cells (Fig. 2t). Among all tested stages, the strongest labelling of the nucleoplasm was observed in the 120-h CDP. In the cytoplasm, a signal was observed particularly around the nucleus, allowing the cytoplasm to be easily distinguished from the cell nucleus. The signal distribution in the population of divided cells cultured for 120 h (Fig. 2u) did not differ significantly from the non-divided cells; however, the divided cells have much more 25S rRNA transcripts especially in the cytoplasm, and the fluorescence in nucleoli is weaker. The cytoplasmic and nucleolar amount of these transcripts increased compared to their level in the 72-h CDP (Fig. 3d, e).

## Discussion

In the process of isolation and the subsequent 120 h of *A. thaliana* mesophyll protoplast culture, fluctuations in the quantity of the active form of RNA POL II, poly(A+) RNA and rRNA were observed. The analysis of the level and distribution of these molecules resulted in establishing a time-space model of the expression of genes transcribed by RNA POL II (poly(A+) RNA) and RNA POL I (25S rRNA) during dedifferentiation and the repeated progression into the mitotic cycle of the cultured cells.

Protoplast isolation results in chromatin decondensation as described in *A. thaliana* (Tessadori et al. 2007), tobacco (Zhao et al. 2001) and *Cucumis sativus* (Ondřej et al. 2009). The relaxation of chromatin is the first step of mesophyll cell dedifferentiation. In the case of *A. thaliana*, the number of chromocenters was decreased, yet no changes in the level of DNA methylation or H3K4 me2 and H3K9 me2 histones, which are a characteristic of condensed chromatin, were observed (Tessadori et al. 2007). A study of the quantity of the *Athila* retroelement (Avivi et al. 2004), as well as that of the introduced silent locus A containing multiple copies of the hygromycin phosphotransferase (*HPT*) gene in *A. thaliana* protoplasts, indicated that chromatin decondensation does not cause an increase in the number of transcripts in the investigated sequences (Tessadori et al. 2007). However, this study does not explain the impact of the changes in chromatin organisation on the overall level of gene expression in protoplasts. Our study is based on quantitative measurements and has revealed an approximate 8.5-fold decrease in the level of transcriptionally active RNA POL II in isolated protoplasts. Our findings show that in *A. thaliana* protoplasts, despite chromatin decondensation, the genome is not strongly activated, but rather the reverse is true that the transcription of RNA POL II genes is reduced. Very similar results were obtained for tobacco protoplasts (Dełeńko and Niedojadło 2011). Consequently, chromatin decondensation in protoplasts may be a result of stress related to the removal of the cell wall, and contrary to a number of examples given in the literature (Müller et al. 2001; Wegel and Shaw 2005; Tittel-Elmer et al. 2010; Sheffield and Furey 2012), decondensation is not connected with an increase in transcription in this particular model. This decrease in POL II transcription activity as shown in the protoplasts might be one of the processes leading to or even being indispensable in the dedifferentiation of plant cells.

Chupeau et al. (2013), in their transcriptional analysis using complete *Arabidopsis* transcriptome microarrays (CATMA), showed that 1728 genes were upregulated in protoplasts. Our results showed that, in protoplasts, the amount of actively transcribing POL II is strongly reduced, and the observed single foci of fluorescence may be the location of transcription of these upregulated genes. The lack of a significant decrease in the quantity of transcripts, despite a decrease in the amount of POL II EF, at this stage, may be related to the impaired maturation and degradation of RNA (Reddy et al. 2013; Wolf and Passmore 2014). However, our results show total RNA POL II transcription activity, including the transcription of protein-coding and non-coding genes, whereas transcriptome analysis on CATMA focuses mainly on protein-coding genes and does not cover RNA POL II transcripts such as miRNA, snRNA, snoRNA and lncRNA, which are poorly represented or completely absent in this system. Therefore, it seems that the problem with estimation of gene expression level in protoplasts requires further studies, probably using additional techniques.

The subsequent investigated stages displayed a clear decrease in the quantity of poly(A+) RNA and 25S rRNA particularly in the cytoplasm. Initially, a gradual decrease in 25S rRNA, which was already visible in the protoplasts, was observed, and the decrease in the number of transcripts of poly(A+) RNA appeared in later stages of culturing, i.e. after 24 h. In the case of poly(A+) RNA, the level in the nucleus did not correlate with the decrease of POL II transcription activity and in no stages was it lower than that in the nuclei from leaf mesophyll cells. Thus, this result is an indication of the presence of mechanisms allowing the accumulation/retention of poly(A+) RNA in the cell nucleus in the isolated and cultured protoplasts. In plant cells that are exposed to heat and ethanol stress conditions, the number of nuclear proteins conjugated with SUMO proteins increases, and poly(A+) RNA accumulates in the cell nucleus (Muthuswamy and Meier 2011). Because hypoxia (Chupeau et al. 2013) and oxidative stress (Ondřej et al. 2010) are among those stresses occurring in protoplasts, the presence of high levels of poly(A+) RNA despite lowered transcription might be a result of stress-related nuclear retention of these transcripts. The RNA retention in the cell nucleus is currently regarded as one of the major stages of gene expression regulation (Boothby and Wolniak 2011; Göhring et al. 2014). Recent findings also suggest that CBs might be the location of nuclear storage of messenger RNA (mRNA) in plant cells (Smoliński and Kołowerzo 2012; Niedojadło et al. 2014 not published). Our study, however, did not reveal a strong accumulation of poly(A+) RNA in CBs in any of the investigated stages, which is an indication that contrary to meiotic cells and meristematic root cells, CBs in the dedifferentiated cells are not domains in the cell nucleus that are involved in mRNA storage.

A clearly marked decrease in poly(A+) RNA was observed in the cytoplasm, with the lowest value observed after 72 h of isolation. At this stage, the poly(A+) RNA transcripts are almost undetectable in the cytoplasm. An increase in the ribonuclease level observed in tobacco protoplasts after 24 h following isolation may be responsible for the process of removing poly(A+) RNA from the cytoplasm (Lazar et al. 1973). This process is most likely to result in removing the translationally active poly(A+) RNA derived from the dedifferentiated mesophyll cells in CDP subjected to dedifferentiation. Next, an increase in poly(A+) RNA in the cytoplasm to a level similar to that in the leaf cells was observed. The differences in the level of poly(A+) RNA presented in this work are most likely a consequence of gene deregulation during the process of protoplast dedifferentiation. During this process, in the case of both *Physcomitrella patens* and *A. thaliana*, the model of gene expression is changed (Xiao et al. 2012; Chupeau et al. 2013), which is a result of genome reprogramming. Transcriptome studies performed on *A. thaliana* found that within a week of protoplast culture, the expression of as many as 5276 genes was changed (Chupeau et al. 2013).

The decrease in the number of 25S rRNA transcripts in the cytoplasm immediately following protoplast isolation and during their 24-h culture resembles the process of ribosome eradication from the cytoplasm during meiosis and the transition from sporophyte to gametophyte during micro- and megasporogenesis in plants (Mackenzie et al. 1967; Dickinson and Heslop-Harrison 1970; Dickinson and Potter 1978). The expression of genes coding for ribosomal proteins also decreases during the isolation and culturing of *P. patens* and *A. thaliana* protoplasts (Xiao et al. 2012; Chupeau et al. 2013). The pronounced decrease in the amount of rRNA in the CDP and protoplasts cytoplasm might reflect the cytoplasm “cleaning” of mesophyll cell ribosomes. It may be a result of ribonucleases RNS1 (AT2G02990) and RNS2 (AT2G39780) activity which mRNA levels increase during protoplast isolation and culture (Chupeau et al. 2013). Ribonuclease RNS2 is involved in the ribosome decay pathway and participates in the housekeeping mechanism that recycles ribosomes during the cell life (Hillwig et al. 2011). Strong protein degradation also likely occurs in the isolated and cultured protoplasts, which is indicated by an increase in the amount of ubiquitin-coding mRNA in *N. tabacum*-isolated protoplasts (Jamet et al. 1990), as well as the fact that the inhibition of the ubiquitin proteasome system makes it impossible for these cells to re-enter the S-phase of the cell cycle (Zhao et al. 2001). Additionally, a *kyp-2 A. thaliana* mutant that is characterised by decreased or totally silenced genes associated with the ubiquitin proteasome system displayed disturbances in callus formation (Grafi et al. 2007).

Therefore, the removal of rRNA first and then poly(A+) RNA from the cytoplasm, as shown by us, is likely a crucial process in plant cells in obtaining totipotency. These processes lead to the removal of ribosomes and protein-coding transcripts derived from mesophyll cells. Hence, following the introduction of a new model of expression enabling a cell fate switch, the newly formed mRNA transcripts are translated on a newly formed translational apparatus.

As shown in our study, the increase in transcripts synthesised by RNA POL I and II at subsequent stages of culturing, after cleaning the cytoplasm, results in cell division. In the nucleolus, the increase in the level of transcription indicated by the increase in rRNA was observed after 24 h following isolation, which consequently resulted in an increase in the level of rRNA in the cytoplasm as early as 72 h after culturing, whereas poly(A+) RNA in the cytoplasm sharply decreased after 72 h and only increased after 120 h following isolation. A study of the transcriptome of the cultured protoplasts of *A. thaliana* displayed an increase in gene transcripts whose products are involved in RNA polymerase activity, the mRNA of ribosomal proteins and nucleolin involved in pre-rRNA processing (Chupeau et al. 2013). Following the transcription initiation of new genes and under hormonal induction, the dedifferentiated cells begin the divisions that lead to re-differentiation.
